# *Myrica rubra* Preharvest Treatment with Melatonin Improves Antioxidant and Phenylpropanoid Pathways During Postharvest Storage

**DOI:** 10.3390/foods14010064

**Published:** 2024-12-29

**Authors:** Jun-Quan Chen, Yun-Shuang Ma, Hejiang Zhou, Rui-Xue Yu, Miao Xiong, Na Yang, Ji-Qiu Wang, Yang Tian, Ling-Yan Su

**Affiliations:** 1 College of Food Science and Technology, Yunnan Agricultural University, Kunming 650201, China; 15125919217@163.com (J.-Q.C.); 13518719921@163.com (Y.-S.M.); zhouhej@ynau.edu.cn (H.Z.); 15912838995@163.com (R.-X.Y.); 19851782063@163.com (M.X.); 18788577093@163.com (N.Y.); 18787938902@163.com (J.-Q.W.); 2 Yunnan Provincial Laboratory of Precision Nutrition and Personalized Manufacturing, Yunnan Agricultural University, Kunming 650201, China; 3 National Research and Development Professional Center for Moringa Processing Technology, Yunnan Agricultural University, Kunming 650201, China; 4 School of Tea and Coffee, Puer University, Puer 665000, China

**Keywords:** melatonin, *Myrica rubra*, pre-harvest treatment, post-harvest storage, antioxidant pathway, phenylpropanoid pathway

## Abstract

*Myrica rubra* is known for its popularity and robust nutritional value. While fresh *Myrica rubra* fruit is a perishable commodity, it has a short post-harvest life and is susceptible to fungal decay after harvest. Melatonin has been reported to delay the aging and quality decline of various fruits and vegetables after harvest. However, the effects of pre-harvest melatonin treatment on the maintenance of post-harvest quality and storage extension of fresh *Myrica rubra* fruit are still unclear. The impact of pre-harvest spraying of melatonin at different concentrations (100 μM, 300 μM, and 500 μM) on the fruit quality of *Myrica rubra* during storage at room temperature or 4 °C was investigated. The results indicated that in the final stage of storage, compared with the control group, different concentrations of melatonin reduced the decay index by 13.0–47.1% and also decreased the weight loss, the content of O_2_^−•^, and the content of malondialdehyde (MDA), respectively. Meanwhile, melatonin increased the content of antioxidants such as superoxide dismutase (SOD), peroxidase (POD), and catalase (CAT), as well as the total polyphenols and flavonoids content. Finally, RNA transcriptome sequencing revealed that melatonin enhanced the antioxidant capacity by increasing the expression of both antioxidant enzymes and changing phenylpropanoid pathway-related genes, therefore maintaining the fresh *Myrica rubra* quality. Our findings uncovered a potent role and mechanism of melatonin in maintaining *Myrica rubra* fruit quality during storage and suggest that pre-harvest melatonin spraying may be a convenient and effective method for prolonging storage and maintaining quality of fruits after picking.

## 1. Introduction

*Myrica rubra*, also called Chinese bayberry, Chinese strawberry, yangmei, and yamamomo, is one of the most important subtropical fruit varieties in China. It is moderately sweet and sour, and rich in nutrients and natural active substances such as phenols. These active substances have anticancer, antioxidant, weight loss, and neuroprotective properties, which make them highly nutritious and therapeutically beneficial [1,2]. *Myrica rubra* ripens in June and July of each year, coinciding with the rainy season. Nevertheless, because of their high tissue moisture content and lack of firm pericarp protection, fresh *Myrica rubra* fruits are vulnerable to mechanical damage during harvesting and transportation [3]. Moreover, the metabolic balance of reactive oxygen species (ROS) is easily disturbed, so that *Myrica rubra*, as a perishable commodity, has a short post-harvest life, can be stored for only 1–2 days, and is susceptible to post-harvest fungal decay at room temperature [1,4].

Excessive ROS are known to cause cellular membrane damage and accelerate senescence and fruit decay [5,6,7]. The ROS balance is maintained by regulating the contents of antioxidants (glutathione, GSH; ascorbate, AsA) and the activity of enzymes involved in ROS metabolism, including superoxide dismutase (SOD), catalase (CAT), glutathione reductase (GR), and peroxidase (POD) [8]. Previous studies have shown that post-harvest inducer treatments could improve the capacity of antioxidants and delay the deterioration of fruit by enhancing ROS scavenger system activity, including reducing superoxide anion generation rate and malondialdehyde (MDA) content, improving the activity of ROS-scavenging enzymes and increasing the levels of non-enzymatic antioxidants, such as total phenolics and anthocyanin [6,9].

In plants, the phenylpropanoid pathway is an important secondary metabolic pathway. It is involved in the synthesis of manifold phenolic compounds, such as flavonoids, coumarins, and lignin [10,11]. Phenylalanine ammonia-lyase (PAL), cinnamic acid 4-hydroxylase (C4H), and 4-coumarate-CoA ligase (4CL) are the three major rate-limiting enzymes in this pathway [12]. PAL functions as the primary regulatory enzyme in phenylpropanoid metabolism. It deaminates phenylalanine to trans-cinnamic acid. Trans-cinnamic acid undergoes hydroxylation to 4-coumarate by C4H activity, followed by the conversion of 4-coumarate to the 4-coumaroyl-CoA by 4CL [10,11]. It plays a key role not only in antioxidant production and disease resistance, but also in plant survival [11,13]. Previous studies have reported that phenylpropanoid pathway accumulation enhances the antioxidant capacity of vegetables and fresh-cut fruits [14,15,16].

Melatonin, also known as N-acetyl-5-methoxytryptamine, a derivative of the essential amino acid tryptophan, was first isolated in the pineal gland by Lerner in 1960 [17]. The recognized therapeutic and health benefits of melatonin may be broad, such as regulating the human circadian rhythm, alleviation of insomnia [18], scavenging free radical species [19,20], immune enhancement and anti-inflammation [21], neuroprotection [20,22,23], anti-aging [24],and anti-cancer [25]. Melatonin was long thought to be a hormone produced only in the pineal glands of animals. However, it was later identified in bacteria [26], fungi [27], insects [28], and plants [29]. Melatonin is also found in many types of foods [30]. It has been implicated in the regulation of seed germination, lateral root development, growth and development, ripening and aging, and the stress response of plants [31,32]. Recent research has shown that melatonin is important for the regulation of development, physiology, and stress response and quality maintenance in fruits and vegetables [33,34]. It was found that melatonin treatment can delay senescence and maintain the quality of jujube [35], plum [36], apple [37], eggplant [38], Hami melon [39,40], fresh-cut Gastrodia elata [41], fresh Gastrodia elata [42], green pepper [43], sweet cherry during storage [44], and water bamboo shoots [45]. However, the effect of melatonin on the quality and the extension of the storage of fresh *Myrica rubra* after harvesting are still unknown.

At present, studies on melatonin in the preservation of vegetables and fruits are mainly focused on post-harvest treatment. In *Myrica rubra* fruit, treatment with melatonin has been scarcely reported to maintain the quality of fruit during storage. The effect of melatonin on the capacity of the antioxidant and phenylpropanoid pathways in *Myrica rubra* fruit has not been investigated yet. In the present study, the impact of pre-harvest melatonin spraying on the storage and quality of *Myrica rubra* during post-harvest storage was explored and RNA transcriptome sequencing was employed to investigate the potential mechanisms of melatonin. Our results provide a reference and theoretical basis for the application of melatonin to *Myrica rubra* fruits and other horticultural fruits before harvest.

## 2. Materials and Methods

### 2.1. Experimental Design

The experimental site was located in Jiajing Park, Kunming City, Yunnan Province, China. Melatonin solutions at concentrations of 100, 300, and 500 μM respectively were dissolved in 5 L of water containing 0.5% Tween. The control group consisted of 5 L water with 0.5% Tween. Sprays were applied once each at the green fruit stage, the color change stage, and one week before harvest. Each concentration of melatonin treatment was applied to three trees. After commercial harvest and ripening, fruits of the same size were harvested without mechanical damage and quickly returned to the laboratory for further physiological and biochemical experiments.

The harvested *Myrica rubra* was carefully placed in polyethylene bags. Each polyethylene bag had ten holes with a diameter of 8 mm. Each group consisted of six bags, and each bag contained 20 fruits (weighing 200 ± 3 g). At the time points of 0, 2, 4, and 6 days at room temperature and 0, 2, 4, 11, 18, and 25 days at 4 °C, indicators of change were measured. Three bags were used for the determination of weight loss and decay index, while the other three bags were used for sampling analysis at the measurement points (at each time point, three fruits were randomly selected from each bag, for a total of nine fruits. After crushing, they were frozen in a −80 °C refrigerator for subsequent biochemical experiments and RNA extraction). Three replicates were used for enzyme activity, four replicates were used for RNA transcriptome sequencing (RNA-Seq), and six replicates were used for real-time PCR (including the four replicates with RNA-Seq).

### 2.2. Determination of Decay Index

The decay index for this study was established as follows: No decay observed; Grade 1: 1–3 minor decay spots, covering approximately 3–5% of the fruit’s surface area; Grade 2: decay covering 25–50% of the fruit; Grade 3: over 50% of the fruit’s area decayed [46].

Decay index = ∑ [(rotten grade × number of fruits of this grade)/(the highest grade of decay × total number of fruits)] × 100%

### 2.3. Determination of Weight Loss, O_2_^−•^ and MDA Contents

We recorded the daily weight of the fruits, and determined the weight loss rate by using the following formula [46]:

Weight loss rate (%) = [(W_0_ − W_t_)/W_0_] × 100%

where W_0_ is the initial weight of the *Myrica rubra* fruits after harvest, and W_t_ is the daily weight during storage.

The rate of O_2_^−•^ production was measured using an adapted hydroxylamine method [47]. A total of 0.5 g of *Myrica rubra* tissue was homogenized in phosphate buffer saline (PBS, pH = 7.8) and centrifuged at 1000× *g* for 10 min at 4 °C, and the supernatant was mixed with equal parts 50 mM PBS and 1M hydroxylamine hydrochloride. After 20 min at 25 °C, 2.0 mL each of 4-aminobenzenesulfonic acid (17 mM) and α-naphthylamine (7 mM) were added, followed by incubation at 30 °C for 30 min. Absorbance at 530 nm was recorded to determine the O_2_^−•^ content, which was calculated as μmol/g.

The content of MDA was detected by the TBA method [48]. Briefly, 0.5 g *Myrica rubra* fruit tissue was homogenized in 2.0 mL of 50 mM phosphate buffer saline (PBS, pH = 7.8), then centrifuged at 1000× *g* for 10 min at 4 °C. The supernatant was mixed equally with TBA solution (6.7 g/L), boiled at 95 °C for 20 min, and then rapidly cooled on ice. The absorbance was measured at 450, 532, and 600 nm, and the MDA content was calculated as μmol/g.

### 2.4. Detecting the Contents of SOD, POD, and CAT

A total of 0.5 g *Myrica rubra* fruit tissue was added to 5.0 mL of sodium phosphate buffer (PBS, pH = 7.8, 50 mmol·L^−1^). The resulting homogenate was ground in an ice bath and centrifuged at 1000× *g* for 10 min at 4 °C.

SOD activity was quantified according to a precise protocol [49]. The reaction mixture contained 0.3 mL each of methionine (130 mM), EDTA (0.1 mM), NBT (0.75 mM), and riboflavin (0.02 mM). We added 0.3 mL enzymatic extract and exposed the solution at 3000 lux for 3 min at 25 °C. After the reaction, NBT photoreduction was measured by absorbance at 560 nm. To measure superoxide radical scavenging efficiency, SOD activity, defined by NBT inhibition, was expressed as U/mg protein.

POD activity was assessed using a modified method protocol [50]. The components mixed were 2.0 mL acetate buffer (0.1 M), 1.0 mL guaiacol (0.25%), 0.5 mL enzyme extract, and 0.1 mL H_2_O_2_ (0.75%). POD activity was measured by absorbance at 470 nm after 5 min at room temperature. One unit is equal to the amount of the enzyme that gives a specific absorbance. This activity has been expressed in units per milligram of protein (U/mg protein).

The activity of catalase (CAT) was measured according to a previously reported method [51] with modifications for accuracy. The absorbance at 240 nm was recorded after 30 s of mixing 0.1 mL of enzyme extract with 2.9 mL of 20 mM hydrogen peroxide using distilled water as a reference. Units per milligram of protein (U/mg protein) was used to express the activity of CAT.

### 2.5. Total Phenolic and Flavonoid Content Determined

Total phenolic content was measured by a Plant Total phenolic kit (adsbio Co., Ltd., Yancheng, China), and flavonoids content was measured by a Plant flavonoid kit (adsbio Co., Ltd., Yancheng, China), according to the manufacturer’s instructions. An EPOCH2 Microplate Reader (BioTek Instruments, Inc., Highland Park, Winooski, VT, USA) was used to detect the absorbance values at 760 nm and 470 nm, respectively, with values expressed as mg/g.

### 2.6. Total RNA Extraction, RNA Transcriptome Sequencing (RNA-Seq) and Data Analysis

For the RNA-Seq analysis, we used samples of *Myrica rubra* fruit from the pre-harvest melatonin-treated group (0, 300 μM) stored at room temperature for 2 days. Each group had four biological replicates. The total RNA of *Myrica rubra* was extracted using the StarSpin Plant RNA Kit (Polysaccharides and Polyphenols-rich) (GenSTAR Co., Ltd., Beijing, China). Biolinker Technology (Co., Ltd., Kunming, China). performedquality control, library construction, and sequencing. The sequencing was carried out using Illumina’s Novaseq6000 (ILMN Co., Ltd., Shanghai, China) with a sequencing strategy of PE150. Raw sequencing reads were trimmed with Trimmomatic v0.39 using “LEADING:3 TRAILING:3 SLIDINGWINDOW:4:15 MINLEN:36”. Clean reads were aligned to the Myrica rubra genome (GCA_003952965.2) using STAR v2.7.3a and processed in bam format. Exon-mapped reads, filtered for <10 expression counts, were normalized using DESeq2’s rlog. This was followed by PCA and differential expression analysis. Adjusted P values (P.adjust < 0.05) identified DEGs. GO and KEGG enrichments were analyzed using clusterProfiler v4.0, visualized using ggplot2 and ComplexHeatmap.

### 2.7. Genes Expression Analysis Using Real-Time Quantitative PCR (qPCR)

Following total RNA extraction, cDNA synthesis was executed using the Hifair^®^III 1st Strand cDNA Synthesis SuperMix Reverse Transcription Kit (Yeasen Biotechnology Co., Ltd., Wuhan, China). qPCR was then performed with gene-specific primers (Table S1) and Hieff UNICON^®^ UniversalBlue qPCR SYBR Green Master Mix (Yeasen Biotechnology Co., Ltd., Wuhan, China). The relative expression levels were determined using the 2^−ΔΔCt^ method with the *Myrica rubra* Actin [52] gene serving as an internal reference gene.

### 2.8. Statistical Analysis

Statistical analyses were performed using GraphPad Prism v9.5 to assess the significance of physiological and biochemical differences between control and melatonin-treated groups of *Myrica rubra*. A two-tailed unpaired Student’s *t* test was used for statistical analysis of the differences between two groups, and two-way *ANOVA* was used for statistical analysis of differences among multiple groups. The data are presented as the means ± SD. *, *p* < 0.05; **, *p* < 0.01; ***, *p* < 0.001; ****, *p* < 0.0001; ns, not significant.

## 3. Results

### 3.1. Pre-Harvest Treatment with Melatonin Reduced Decay Index and Weight Loss of Myrica rubra Fruits

**Figure 1:**
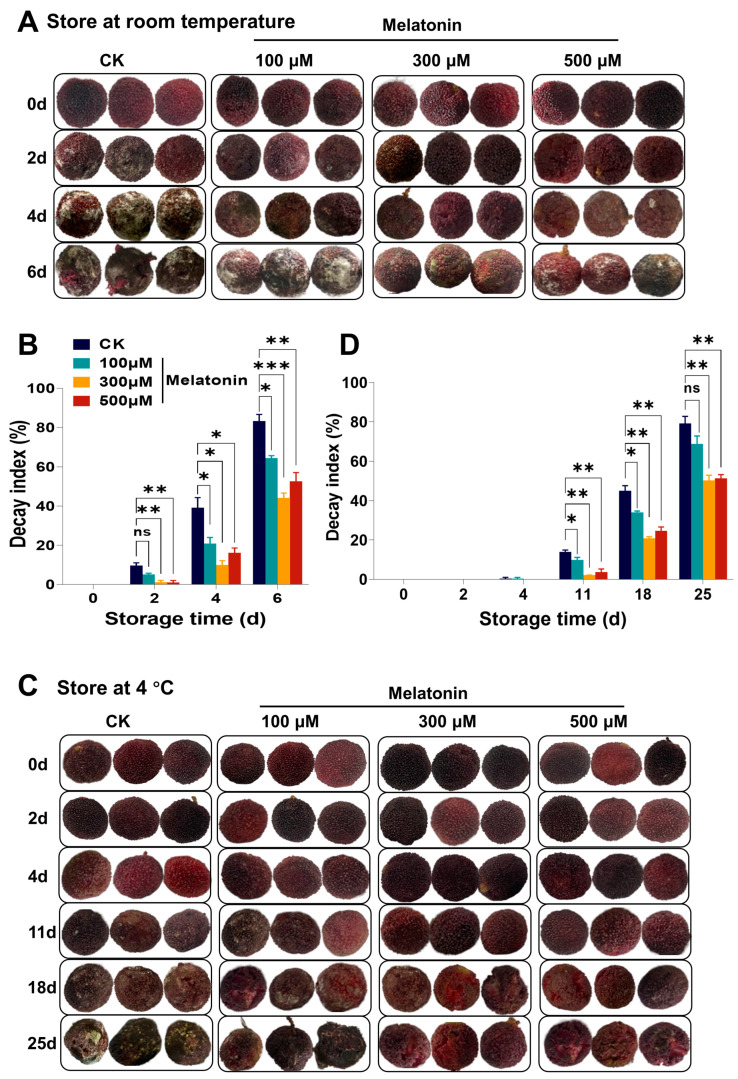
Effects of pre-harvest melatonin treatment (0, 100, 300, 500 μM) on direct characteristics and decay rate of *Myrica rubra* fruits during storage at room temperature and 4 °C. (**A**) Physical map of pre-harvest melatonin treatment (0, 100, 300, 500 μM) groups on *Myrica rubra* fruits stored at room temperature for 0–6 days. (**B**) Decay rate of *Myrica rubra* fruits stored at room temperature for 0–6 days. (**C**) Physical map of pre-harvest melatonin treatment (0, 100, 300, 500 μM) groups on *Myrica rubra* fruits stored at 4 °C for 0–25 days treated with pre-harvest melatonin (0, 100, 300, 500 μM) (n = 3 biological replicates). (**D**) Decay rate of *Myrica rubra* fruits stored at 4 °C for 0–25 days. All results are presented as mean ± SD. Group differences were analyzed by two-way repeated-measures *ANOVA*. *, *p* < 0.05; **, *p* < 0.01; ***, *p* < 0.001; ns, not significant.

*Myrica rubra* fruits do not keep well, and their storage capacity is short at room temperature [4]. Under room temperature storage, the *Myrica rubra* fruits started to show rot on the second day, and on the fourth day, the rot was severe; molds were spread over the whole fruit surface in large quantities. On the sixth day, *Myrica rubra* rot was as high as 83.24%, representing a complete loss of commercial value in the control fruits (Figure 1A,B). *Myrica rubra* fruits stored at 4 °C showed decay on 11th day, and the decay rate was 79.15% in the fruits from the 4 °C control group stored for up to 25 days, losing their commercial value (Figure 1C,D). Rot appeared on the fourth day of storage at room temperature in three pre-harvest melatonin (100, 300, 500 μM) treatment groups (Figure 1A); on the sixth storage day, the rate of rot was 64.27%, 44.05%, and 52.52% (Figure 1B), respectively. Under low-temperature storage, the decay index of *Myrica rubra* fruits was 68.87%, 50.20%, and 51.30% on the 25th day, respectively, across three melatonin doses (100, 300, 500 μM) groups (Figure 1D), with a large number of cracked fruits appearing (Figure 1C). The dose of 300 μM melatonin had the best effect on preventing *Myrica rubra* fruits from decaying during storage.

**Figure 2:**
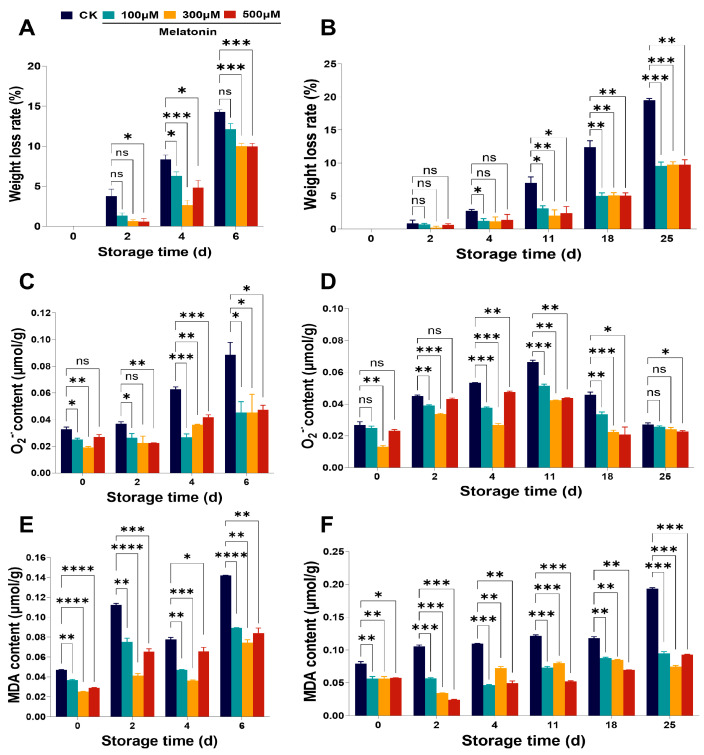
Effect of pre-harvest melatonin treatment (0, 100, 300, 500 μM) on weight loss and O_2_^−•^ and MDA content of *Myrica rubra* fruits during storage at room temperature and 4 °C. Pre-harvest melatonin treatment (0, 100, 300, 500 μM) rescued the increased weight loss (**A**,**B**), O_2_^−•^ content (**C**,**D**), and MDA content (**E**,**F**) of *Myrica rubra* fruits stored at room temperature for 0–6 days and 4 °C for 0–25 days (n = 3 biological replicates). All results are presented as mean ± SD. Group differences were analyzed by two-way repeated-measures *ANOVA*. *, *p* < 0.05; **, *p* < 0.01; ***, *p* < 0.001; ****, *p* < 0.0001; ns, not significant.

Next, we evaluated the impact of melatonin on the weight loss of *Myrica rubra* fruits during storage. Under storage conditions both at room temperature and at 4 °C, the fruits of *Myrica rubra* showed an increasing trend of weight loss across the whole storage period in both the control group and in the group treated with melatonin (100, 300, 500 µM) before harvesting (Figure 2A,B). Pre-harvest treatment with melatonin significantly suppressed the weight loss rate of *Myrica rubra* fruits over storage days 2 to 6 under room temperature (Figure 2A) and from days 4 to 25 under 4 °C (Figure 2B), respectively. These findings indicated that treatment with melatonin before harvest notably reduced the decay index and weight loss of *Myrica rubra* fruits stored at both ambient temperature and 4 °C. Note that melatonin treatment before harvest had no effects on the hardness (Figure S1A,B) and total soluble solids (Figure S1C,D) of *Myrica rubra* fruits.

### 3.2. ROS Metabolism of Myrica rubra Fruits During Storage Could Be Affected by Pre-Harvest Treatment with Melatonin

The metabolic balance of ROS is important for fruits senescence and decay [5,6,7]. We found that the O_2_^−•^ content, one of the major ROS, of *Myrica rubra* fruits in the control group steadily increased during storage at room temperature (Figure 2C). During storage at 4 °C, the O_2_^−•^ content increased at first (days 2 to 11) and then decreased (days 11 to 25) in *Myrica rubra* fruits in the control group (Figure 2D). Similar results were observed in *Myrica rubra* fruits treated with melatonin (100, 300, 500 μM), but the O_2_^−•^ content was markedly lower than in the control fruits at all storage times, both at room temperature and 4 °C (Figure 2C,D).

The level of malondialdehyde (MDA), the end product of lipid peroxidation in cells, is an indicator of the level of lipid oxidation in plants [53]. Our results showed an increasing trend of MDA content in *Myrica rubra* fruits stored in both the control and melatonin (100, 300, 500 μM) treatment groups (Figure 2E,F). However, pre-harvest treatment with melatonin (100, 300, 500 μM) markedly reduced the MDA content at the start of storage and throughout the whole storage period as compared to the control group (Figure 2E,F). The results showed that pre-harvest treatment with melatonin (100, 300, 500 μM) could eliminate the O_2_^−•^ and MDA contents of *Myrica rubra* fruits, clear the excess ROS, inhibit membrane lipid peroxidation, and maintain cell membrane stability, which was of great significance for keeping the fruits fresh.

### 3.3. Pre-Harvest Treatment with Melatonin Increased the Antioxidant Enzymes Activities in Myrica rubra Fruits

**Figure 3:**
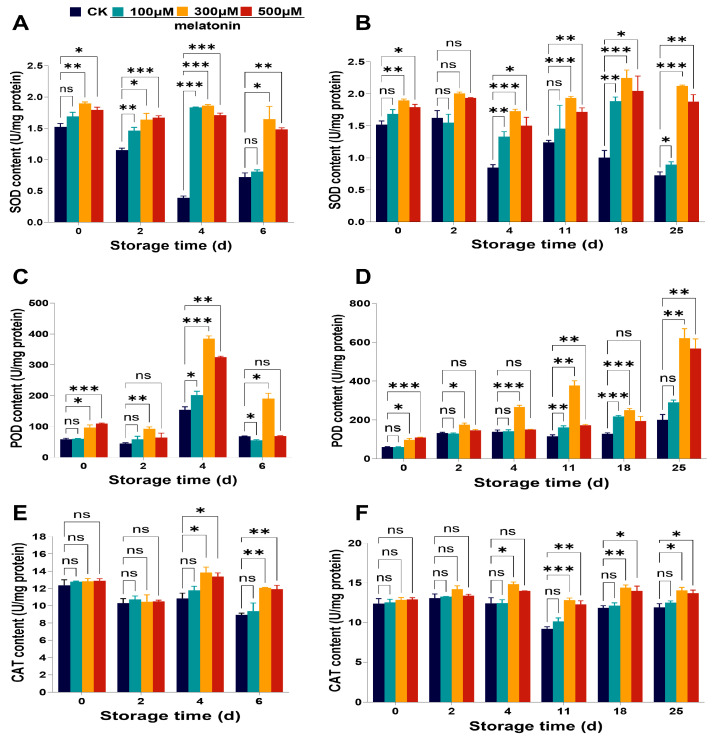
Changes in antioxidant enzymes activities of *Myrica rubra* fruits with pre-harvest melatonin (0, 100, 300, 500 μM) spraying. Pre-harvest melatonin (0, 100, 300, 500 μM) spraying increased the activity of SOD (**A**,**B**), POD (**C**,**D**), and CAT (**E**,**F**) in *Myrica rubra* fruits during storage at room temperature for 0–6 days and 4 °C for 0–25 days (n = 3 biological replicates). All results are presented as mean ± SD. Group differences were analyzed by two-way repeated-measures ANOVA. *, *p* < 0.05; **, *p* < 0.01; ***, *p* < 0.001; ns, not significant.

To investigate whether the influences of melatonin on the metabolism of ROS were related to antioxidant enzymes, we investigated the key antioxidant enzyme activities, such as SOD, POD, and CAT activities. With an extension in storage time, the content of SOD decreased in *Myrica rubra* fruits stored at room temperature for 0–6 days and 4 °C for 0–25 days (Figure 3A,B). Our results showed that the SOD activity was significantly increased in *Myrica rubra* fruits with pre-harvest melatonin (300, 500 μM) treatment on day 0, and a higher SOD activity level throughout the entire storage period was found in *Myrica rubra* fruits with melatonin treatment compared to control fruits (Figure 3A,B). The POD activity (Figure 3C,D) in *Myrica rubra* fruits was significantly increased in the pre-harvest melatonin (300, 500 μM) treatment group at day 0. The activity of POD for the control and melatonin (100, 300, 500 μM) treatment groups exhibited similar trends, continuously increasing, with the maximum activity reached on day 4 at room temperature (Figure 3C). POD activity demonstrated an increased tendency in *Myrica rubra* fruits stored at 4 °C for 0–25 days (Figure 3D). Compared to control fruits, pre-harvest treatment with melatonin seemed to determine a higher POD activity in *Myrica rubra* fruits throughout the entire storage period, but the pre-harvest 100 μM melatonin treatment group had less influence (Figure 3C,D). During storage, all fruits from both the pre-harvest treatment with melatonin (100, 300, 500 μM) and control groups demonstrated a progressive decrease in CAT activity over the first 2 days under room temperature conditions (Figure 3E) and 11 days under 4 °C (Figure 3F), respectively. Notably, the pre-harvest melatonin (300, 500 μM) treatment significantly increased the CAT activity on days 4 to 6 (room temperature) and days 11 to 25 (4 °C) compared to control fruits (Figure 3E,F). Consistent with previous studies [6,9], we observed that pre-harvest spraying of melatonin was beneficial in improving the activities of key antioxidant enzymes, such as SOD, POD, and CAT.

### 3.4. Pre-Harvest Melatonin Treatment Increased the Contents of Antioxidant Compounds in Myrica rubra Fruits

**Figure 4:**
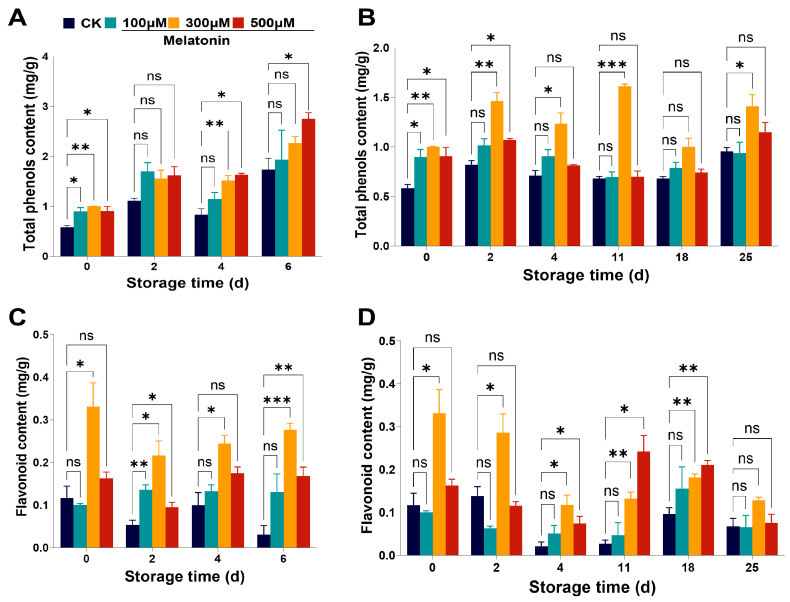
Changes in the content of total phenols and flavonoids in *Myrica rubra* fruits with pre-harvest melatonin (0, 100, 300, 500 μM) spraying. Pre-harvest melatonin (0, 100, 300, 500 μM) spraying increased the content of total phenols (**A**,**B**) and flavonoids (**C**,**D**) in *Myrica rubra* fruits during storage at room temperature for 0–6 days and 4 °C for 0–25 days (n = 3 biological replicates). All results are presented as mean ± SD. Group differences were analyzed by two-way repeated-measures *ANOVA*. *, *p* < 0.05; **, *p* < 0.01; ***, *p* < 0.001; ns, not significant.

As secondary plant metabolites, phenols and flavonoids have strong antioxidant properties and modify the color and flavor of fruits [54]. We next tested the effect of pre-harvest treatment with melatonin (100, 300, 500 μM) on the total phenols and flavonoids content. Our results showed that the total phenolic content was significantly increased in *Myrica rubra* fruits in the pre-harvest melatonin (100, 300, 500 μM) treatment groups on day 0 (Figure 4A,B). With the extension of storage time, the total phenols content began to increase from day 2 in *Myrica rubra* fruits with pre-harvest melatonin treatment, both stored at room temperature and low temperature, compared with the control fruits (Figure 4A,B). The flavonoids content decreased with the extension of storage time in *Myrica rubra* fruits stored at room temperature for 0–6 days and 4 °C for 0–25 days (Figure 4C,D). These effects were shown to be rescued by pre-harvest treatment with melatonin (300, 500 μM). These results indicate that pre-harvest treatment with melatonin may increase the quality of *Myrica rubra* fruits by activating antioxidant systems, including enzymatic and non-enzymatic systems (the content of phenols and flavonoids). The increased content of phenols and flavonoids induced by melatonin would account for the delay in the *Myrica rubra* post-harvest decay process and fruit quality maintenance during storage.

### 3.5. Pre-Harvest Melatonin Treatment Maintains Myrica rubra Quality via Antioxidant Pathway and Phenylpropanoid Pathway

**Figure 5:**
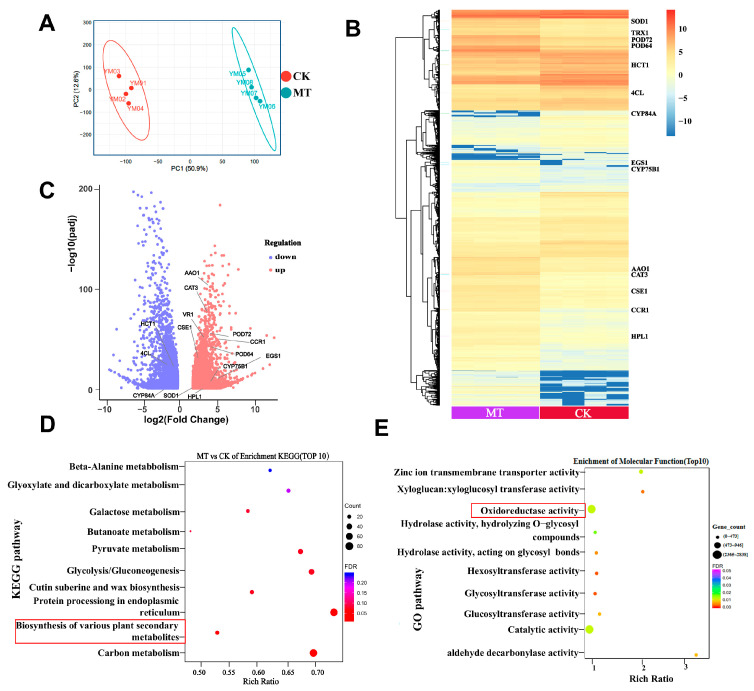
Transcriptomic profiling of *Myrica rubra* fruits with or without pre-harvest melatonin (300 μM) treatment. (**A**) Principal component analysis (PCA) of *Myrica rubra* fruits with or without pre-harvest melatonin treatment (300 μM). PCA was performed based on the expression values of all expressed genes, and each point represented a sample. (**B**,**C**) Heatmaps (**B**) and volcano plot (**C**) showing the differentially expressed genes in *Myrica rubra* fruits between pre-harvest melatonin treatment and control and groups. (**D**,**E**) KEGG pathway enrichment analysis (**D**) and GO biological processes analyses (**E**) of differentially expressed genes in the *Myrica rubra* fruits with and without pre-harvest melatonin treatment.

To explore the molecular mechanisms underlying melatonin pre-harvest treatment-attenuated post-harvest decay and maintained quality of *Myrica rubra* fruits, RNA-seq analyses were performed on *Myrica rubra* fruits from the pre-harvest melatonin treatment and control groups. A clear difference between the *Myrica rubra* fruits from the two groups was revealed by principal component analysis (Figure 5A). The differentially expressed genes between two groups revealed a clearly different signature in *Myrica rubra* fruits from each group, as shown by heatmaps (Figure 5B). We detected a greater number of upregulated and downregulated genes in *Myrica rubra* with pre-harvest melatonin treatment than in the control group (Figure 5B,C). Among these differentially expressed genes, mannan endo-1, 4-beta-mannosidase 7 (*MAN7*), serine carboxypeptidase-like 18 (*SCPL18)*, glutathione S-transferase F11 (*GSTF11)*, and protein disulfide-isomerase (*PDI1*) exhibited significantly increased expression in the Myrica rubra fruits with pre-harvest melatonin treatment (Table S2). We selected the top ten up-regulated and down-regulated genes to validate, and found that the pattern of gene expression was completely matched to the results from RNA-Seq (Figure S2), suggesting that the experimental system was feasible. GO and KEGG enrichment analysis revealed that the activity of several signaling pathways, including the antioxidant and phenylpropanoid pathways, were upregulated in the pre-harvest melatonin treatment group compared to the control group (Figure 5D,E).

**Figure 6:**
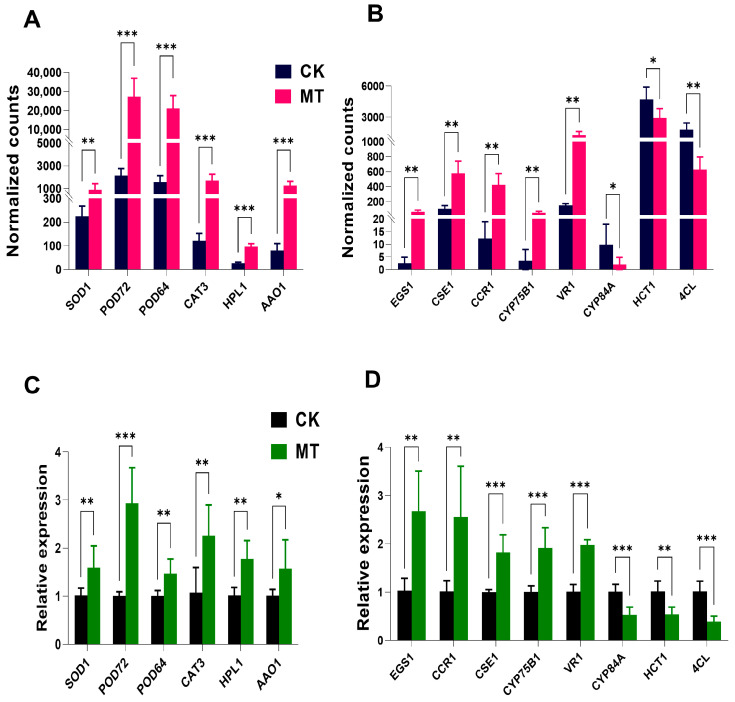
Pre-harvest melatonin (300 μM) treatments altered the gene expression pattern of antioxidant pathways, the phenylpropanoid pathways, and differential genes related to total phenols and flavonoids in *Myrica rubra* fruits. (**A**,**B**) Bar graphs showing the mRNA expression levels of the selected genes of the antioxidant pathway (**A**), the phenylpropanoid pathway, and differential genes related to total phenols and flavonoids (**B**) in the *Myrica rubra* fruits from RNA-Seq (n = 4 biological replicates). (**C**,**D**) Validation of the selected gene expressions in the antioxidant pathway (**C**), phenylpropanoid pathway, and differential genes related to total phenols and flavonoids (**D**) in the *Myrica rubra* fruits by using real-time quantitative PCR (n = 6 biological replicates). Group differences were analyzed by a two-tailed unpaired Student’s *t* test. The data are presented as the means ± SD. *, *p* < 0.05; **, *p* < 0.01; ***, *p* < 0.001.

Among these differentially expressed genes, six genes related to the antioxidant pathway, six genes related to the phenylpropanoid pathway, and two genes related to the synthesis of phenols and flavonoids were excavated that exhibited significantly increased expression in the *Myrica rubra* fruits with pre-harvest melatonin treatment (Table S3). Many antioxidant pathway genes were up-regulated (Figure 6A). The expression of eugenol synthase (*EGS1*), caffeoyl shikimase (*CSE1*) and cinnamoyl coenzyme a reductase (*CCR1*) was up-regulated; ferulate-5-hydroxylase (*CYP84A*), *4CL*, and shikimate O-hydroxycinnamoyltransferase (*HCT1*) were down-regulated in the phenylpropionate pathway (Figure 6B and Figure S3). Then, we used qPCR to further validate the mRNA expression levels of related genes in the *Myrica rubra* fruits with increased sample numbers. Indeed, we observed that the pre-harvest melatonin treatment altered the expression of genes in the antioxidant pathway, such as superoxide dismutase [Mn](*SOD1*), peroxidase 72 (*POD72*), peroxidase 64 (*POD64*), catalase isozyme 3 (*CAT3*), hydroperoxide lyase (*HPL1*), L-ascorbate oxidase (*AAO1*) (Figure 6C), and in the phenylpropanoid pathway, such as *EGS1, CSE1, CCR1, CYP84A, 4CL*, and *HCT1* (Figure 6D). The total phenols and flavonoids content in *Myrica rubra* fruits within the pre-harvest melatonin spraying groups increased compared to the control fruits (Figure 4). The flavonoid 3’-monooxygenase (*CYP75B1*) and vestitone reductase (*VR1),* which are involved in phenols synthesis and flavonoid synthesis, were significantly increased by pre-harvest melatonin treatment in *Myrica rubra* fruits, exhibiting higher incidence than in the control group (Figure 6B,D). These data suggest that pre-harvest melatonin treatment maintains *Myrica rubra* quality during storage through multiple signaling pathways, including the antioxidant and phenylpropanoid signaling pathways.

## 4. Discussion

One of the major constraints on the development of the *Myrica rubra* industry is the short post-harvest storage life of fresh *Myrica rubra*. During storage after harvest, *Myrica rubra* fruit deteriorates through oxidation and dehydration [55]. To date, much of the related research has focused on the post-harvest preservation of *Myrica rubra*, but little work has been conducted on extending the storage of *Myrica rubra* through pre-harvest treatment. Melatonin plays an important role in prolonging the storage of fruit. Previous studies have shown that melatonin treatment delays the hardness decline of kiwifruit [56,57], attenuates the post-harvest decay of strawberry fruits, and maintains the nutritional quality [58] and increases the antioxidant potential of sweet cherries [59], lemons [60], carambola fruit [61], and jackfruit bulbs [62]. Recent research has shown that pre-harvest melatonin spraying can improve the fruit quality of ‘Yuluxiang’ pear [3] and sweet cherry [50]. We found that pre-harvest treatment of *Myrica rubra* fruits with melatonin was effective in reducing post-harvest water loss, delaying fruit ripening and senescence.

ROS are the major mediators of oxidative damage and the aging process in plants, including H_2_O_2_, O_2_^−•^, and OH^−^ [46]. They are generated from the normal metabolism of cells and scavenged by antioxidant enzymes and antioxidants (such as flavonoids, carotenoids, tocopherols, phenolics, and ascorbic acid) [46,50]. Previous studies have found that melatonin increases the activities of antioxidant enzymes, the contents of non-enzymatic antioxidants, and related gene expression during fruits storage [50,59,63,64]. Melatonin pre-harvest treatment led to lower O_2_^−•^ content, higher activities of SOD, CAT, and POD, and increased phenolics and anthocyanins concentrations in *Myrica rubra* fruit at the harvest point and during storage time compared to controls (Figure 3 and Figure 4) at room and low temperatures. Meanwhile, the increased mRNA expression of antioxidant enzyme genes (*SOD1, POD72, POD64, CAT3, HPL1, AAO1)* was also observed in the melatonin treatment group. Thus, the pre-harvest application of melatonin leads to an increase in the ROS elimination systems of Myrica rubra, contributes to the delay of post-harvest senescence and decay processes and extends the storage of fruits.

The phenylpropanoid pathway plays important roles in the growth and development of plants and their response to external stresses, among other things [65]. Up-regulated expression of genes involved with lignin synthesis in alfalfa (*Medicago sativa* L.) under stress conditions was found to support enhanced lignin production, resulting in increased antioxidant enzyme activity to defend against oxidative damage [66]. Previous research has shown that treatment with melatonin increases the expression and activities of *PAL*, *4CL*, *CSE*, and *CCR*, which enhances antioxidant capacity and disease resistance in blueberry [67], litchi [68], cherry tomato [50,63], tomato fruits [69], fresh-cut G. elata [41], and pomegranate fruits [70]. In this study, both the activity and mRNA expressions of *EGS1, CSE1, CCR1, CYP84A*, *4CL*, and *HCT1* were observed to be significantly increased in *Myrica rubra* fruits with pre-harvest melatonin treatment. These enhancements may indirectly lead to elevated levels of phenolic compounds, and thus the expression level of phenylpropanoid pathway was increased, which may contribute to the elevated disease resistance and antioxidant capacity of *Myrica rubra*.

Melatonin had no significant inhibitory effect on *Myrica rubra* fungi such as *Alternaria Nees*, *Botrytis cinerea,* and *Candida* sp. (Figure S4). Therefore, we envisage that the melatonin-mediated disease resistance of *Myrica rubra* fruits may be achieved through direct or indirect regulation of the antioxidant systems, cell wall structural stability, and plant hormone metabolism, rather than having a direct influence on the pathogen. As a class of signaling molecules synthesized naturally, phytohormones play a novel role in the regulation of plant development, physiology, and adaptation to environmental stimulation [71]. Consistent with a previous study [72], pre-harvest melatonin spraying increased the expression of transcription factor MYB26 (*MYB26*) and down-regulated mitogen-activated protein kinase 17 (*MPK17),* LysM domain receptor-like kinase 3 (*LYK3)*, and Class V chitinase (*ChiC)* genes which are involved in gibberellin, abscisic acid, and jasmonic acid signaling in *Myrica rubra* fruits. This suggests that melatonin may delay fruit failure by reducing the biosynthesis and signaling of these phytohormones.

Note that the *Myrica rubra* fruit cracked during storage with pre-harvest spraying of melatonin. Phenylpropanoid biosynthesis can produce more than 8000 secondary metabolites [73]. These secondary metabolites can inhibit the growth of pathogens and limit pathogen invasion by strengthening and protecting plant cell walls [74]. The cell wall maintains cellular shape and provides a link between internal and external factors involved in abiotic and biotic stresses [75]. The xyloglucan endotransglucosylases/hydrolases (*Xth*) gene family is involved in regulating plant cell wall structural functions [76]. Fracture-resistant tomatoes may exhibit greater resistance to osmotic stress with down-regulation of *Xth* genes, which strengthen the cell wall during water stress [77]. In our study, we observed the down-regulation of bases xyloglucan endotransglucosylase/hydrolase protein 22 (*XTH22*) and xyloglucan endotransglucosylase/hydrolase protein 23 (*XTH23*) (Figure S2), which are related to cell walls in melatonin induction, and favored an increase in *Myrica rubra* cell wall resistance to external stress.

## 5. Conclusions

**Figure 7:**
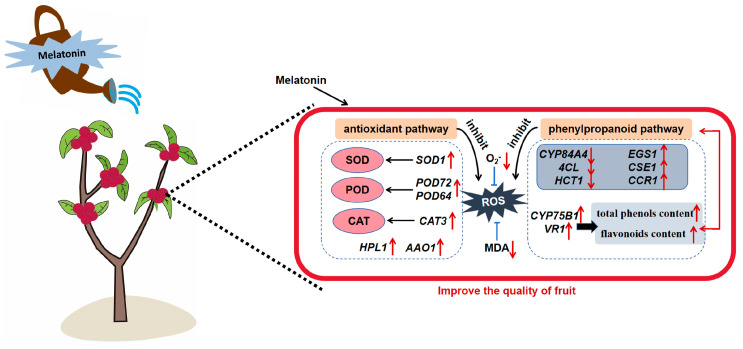
Schematic of potential mechanism underlying the effects of pre-harvest melatonin treatment on *Myrica rubra* fruits quality. Melatonin pre-harvest treatment attenuated post-harvest decay and maintained the quality of Myrica rubra fruits by increasing the activities and expression of antioxidant enzymes and enhancing the phenylpropanoid pathway to promote total phenols and flavonoids accumulation. (Red upward arrows indicate gene upregulation, red downward arrows indicate gene downregulation, black arrow direction indicates the direction of transformation, blue T-shaped arrows indicate repression, and red two-way arrows indicate interaction).

In summary, melatonin pre-harvest treatment effectively extends the post-harvest life and enhances the quality of *Myrica rubra* fruits. The potential mechanisms involve increasing antioxidant capacity and regulating the phenylpropanoid pathway (Figure 7). Pre-treatment with melatonin significantly decreases O_2_^−•^ and MDA content, decay index, and weight loss and inhibits microorganism growth, and thus improves fruit safety. Moreover, it raises antioxidant enzyme (SOD, POD, CAT) activity to maintain post-harvest quality. The phenylpropanoid pathway activation by pre-harvest melatonin treatment increases total phenolics and flavonoids contents, enhancing antioxidant capacity. Pre-harvest melatonin spraying is a promising method to reduce post-harvest fruit rot, and our study deepens the understanding of the mechanisms of pre-harvest melatonin treatment on *Myrica rubra* post-harvest physiology and storage.

## Data Availability

The original contributions presented in the study are included in the article/Supplementary Materials; further inquiries can be directed to the corresponding authors.

## Supplementary Materials

The following supporting information can be downloaded at: https://www.mdpi.com/article/10.3390/foods14010064/s1, Table S1. Primer pairs for quantifying mRNA expression level of interested genes. Table S2. TOP15 differentially expressed genes (DEGs). Table S3. Genes related to antioxidant pathway, phenylpropanoid pathway, total phenols and flavonoids content. Figure S1. Effect of pre-harvest melatonin treatment (0, 100, 300, 500 μM) on hardness and total soluble solids content (TSS) of *Myrica rubra* fruits during storage at room temperature and 4 °C. Figure S2. Verification of the top 10 genes that showed a change in expression with pre-harvest melatonin treatments in *Myrica rubra* fruits. Figure S3. The effects of pre-harvest melatonin treatment on phenylpropanoid pathway in *Myrica rubra* fruits. Figure S4. Effect of the in vitro treatment with melatonin (0, 100, 300, 500 μM) on the morphology of fungal colonies in PDA medium and on the diameter of fungal colonies on *Myrica rubra* fruits. Reference [78] is cited in Supplementary Materials.

## Abbreviations

AsA	Ascorbate
APX	Ascorbate peroxidase
AAO1	L-ascorbate oxidase
At1g02270	Uncharacterized calcium-binding protein At1g02270
CAT3	Catalase isozyme 3
C4H	Cinnamic acid 4-hydroxylase
CSE1	Caffeoyl shikimase
CCR1	Cinnamoyl coenzyme a reductase
CYP84A	Ferulate-5-hydroxylase
CYP75B1	Flavonoid 3’-monooxygenase
ChiC	Class V chitinase
EGS1	Eugenol synthase
GR	Glutathione reductase
GSH	Glutathione
GSTF11	Glutathione S-transferase F11
HPL1	Hydroperoxide lyase
HCT1	O- hydroxycinnamoyltransferase
LYK3	LysM domain receptor-like kinase 3
Melatonin	MT
MDA	Malondialdehyde
MAN 7	Mannan endo-1,4-beta-mannosidase 7
MPK17	Mitogen-activated protein kinase kinase kinase 17
MYB26	Transcription factor MYB26
POD72	Peroxidase 72
POD64	Peroxidase 64
PAL	Phenylalanine ammonia-lyase
PDI1	Protein disulfide-isomerase
PBL16	Probable serine/threonine-protein kinase PBL16
ROS	Reactive oxygen species
SOD	Superoxide dismutase
SOD1	Superoxide dismutase [Mn], mitochondrial
SCPL18	Serine carboxypeptidase-like 18
VR1	Vestitone reductase
XTH23	Probable xyloglucan endotransglucosylase/hydrolase protein 23
XTH22	Xyloglucan endotransglucosylase/hydrolase protein 22
4CL	4-coumarate-CoA ligase
