# Supplementary material for: Myrica rubra Preharvest Treatment with Melatonin Improves Antioxidant and Phenylpropanoid Pathways During Postharvest Storage
Source: Foods. 2024 Dec 29;14(1):64. doi: 10.3390/foods14010064 (PMC11719693; doi:10.3390/foods14010064)
Supplement: Supplementary file 1 [file foods-14-00064-s001.zip › foods-3368711-supplementary.pdf]

Online Methods and Data Supplements

Materials and Methods

Determination of hardness and Total soluble solids (TSS) content

Fruit hardness: Fruit hardness was analyzed using a GY-1 fruit hardness tester (topyiqi Co., Ltd. Zhejiang, China). The hardness was expressed as 10^5Pa.Three biological replicates were performed

TSS content: determined using a hand-held digital display refractometer (Wangchen Instrument Co.,Ltd., Chengdu,China).Three biological replicates were performed

Isolation and purification of pathogens

According to the previous study (Qu, Wu, Ba, Ma, Ji, & Cao, 2022), 50 µL of Myrica rubra culture solution was taken into 950 µL of sterile water, mixed well, 10ul of the bacterial solution was applied to PDA medium with different concentrations of melatonin(100, 300 and 500µM). And cultivated in the incubator at a constant temperature of 28℃ for 3~5 d. After the pathogen isolation and purification of decayed Myrica rubra using the method of single-spore isolation, the strains obtained from the isolation were back-joined according to the law of Kochs and identified as pathogenic bacteria (Lin et al., 2019). The growth condition was observed every day and the growth diameter (cm) were calculated by cross crossing method.

Supplementary References

Lin, Y., Fan, L., Xia, X., Wang, Z., Yin, Y., Cheng, Y., & Li, Z. (2019). Melatonin decreases resistance to postharvest green mold on citrus fruit by scavenging defense-related reactive oxygen species. *Postharvest Biology and Technology, 153*, 21-30. https://doi.org/10.1016/j.postharvbio.2019.03.016.

Qu, G., Wu, W., Ba, L., Ma, C., Ji, N., & Cao, S. (2022). Melatonin Enhances the Postharvest Disease Resistance of Blueberries Fruit by Modulating the Jasmonic Acid Signaling Pathway and Phenylpropanoid Metabolites. *Frontiers in Chemistry, 10*, 957581-957591. https://doi.org/10.3389/fchem.2022.957581.

Supplementary Tables

Supplementary Table S1. Primer pairs for quantifying mRNA expression level of interested genes

**Primer**	**Sequence (5'-3')**	**Gene**	**Product length (bp)**
**Primers for antioxidant systems genes**			
r*SOD1* F	ATGGGTGTGGCTTGGTCTGG	*SOD1*	113
r*SOD1* R	CAATACCGAGCAAAGGAACTAAATGTG		
r*POD72* F	TTCTCACCAAGTTCAAGCGACAAG	*POD 72*	114
r*POD72* R	GGTTGTAAAGCCTCTGCCTGAAG		
r*POD64* F	TCTTGGGTTCGCTCATTGTTCATC	*POD64*	103
r*POD64* R	TAGGCTGGCTGCGAAGGATG		
r*CAT3* F	ATGTGGTTCCTGGTATCTACTACTCC	*CAT3*	89
r*CAT3* R	TCCAAGACGGTGCCTGTGAG		
r*HPL1* F	CTACATCGTCCCTCTCCAAAAGTTC	*HPL1*	120
r*HPL1* R	AGCCAGCGGTCAAGCATAATATAAC		
r*AAO1* F	GATAGAGTCTGCCACCAACGATAAC	*AAO1*	115
r*AAO1* R	TCCATCCTGCCACGAGTTCC		
**Primers for phenylpropanoids biosynthesis genes**			
r*EGS1*F	GGAACTGAACGAGCACGAGAAG	*EGS1*	109
r*EGS1* R	GGCGTCTATGATCTTGAGTTGGTC		
r*CSE1* F	CTGAATGAGATCGCCTCGAAGAAC	*CSE1*	87
r*CSE1* R	AAGATGTTGTTGAGCCTCCAAGAAG		
r*CCR1* F	AACTGTGGAGCCAATTCTGAAGC	*CCR1*	95
r*CCR1* R	CAGTCGCAGTATCTCTAACATCAACC		
r*CYP84A* F	ACTGTGGCGTCAGCGATAGAG	*CYP84A*	93

r*CYP84A* R	CACTACTTCGGAGAGTTCTTGTTGG		
r*4CL1* F	CCCGAGGAAGAAGTGATTGAACC	*4CL1*	115
r*4CL1* R	TGACCACAGCAGAAGCAGAGG		
r*HCT1* F	TACACTTCCGCCGTTCATTGACAG	*HCT1*	87
r*HCT1* R	GCAGGTTGGTACTCGATGTGGTG		
**Primers for total phenols and flavonoid content genes**			
r*CYP75B1* F	AATGGGTAATGGCGGAGTTGATG	*CYP75B1*	111
r*CYP75B1* R	TGGGCAAATGGGATTCTTCTACTATG		
r*VR1* F	GAGGACTGTGAAGCGAGTTGTG	*VR1*	93
r*VR1* R	CTCCAGAAGCTCTCATCCATTGC		
**Primers for up-regulated genes**			
r*At1g02270* F	CATACTCTGCGGTGACTGGAATGG	*At1g02270*	146
r*At1g02270* R	CCCTGTGGTTTCGGTGGCTAAC		
r*MAN7* F	ATCTCGTCTGCGTTTGAAGAAGC	*MAN7*	81
r*MAN7* R	TCTATACCCTCCGTCACTGAAAGC		
r*ICE1* F	GGTCCGTGGTTCCGAAGATTAGC	*ICE1*	148
r*ICE1* R	GTGGTAGGCGTCAACGAAGAGC		
r*NPF2.11* F	GAAGGACAACGGCTCTAACCAAG	*NPF2.11*	104
r*NPF2.11* R	GGCTAGTGCGAGTTGAGGAATC		
r*GSTF11* F	GAGCCATCTTCCGTGCGTTAG	*GSTF11*	141
r*GSTF11* R	TAACCAGCAAGCGTCAGTAACTTC		
r*PHT1-7* F	AAATCCCGCTTCTGTTATGGCTAC	*PHT1-7*	127
r*PHT1-7* R	GGCTCCACGAGTCTTCTTGTTC		
r*PDI1* F	GCCGAACTTGGATGCCGATCAG	*PDI1*	112

r*PDI1* R	CACAGGCTCGTTGTTCTCTTCAGG		
r*PBL16* F	AGAGCAGAACCTAGCCGATTGG	*PBL16*	122
r*PBL16* F	GCATACCAGCCTTGTGAACTCC		
r*ILR1* F	CACCAAGATAGAGGACCAGCAAAC	*ILR1*	101
r*ILR1* R	AACCGCATCTCACCTTACACAAC		
r*SLC47A* F	AGGACTCTTGCTCGGCTACG	*SLC47A*	147
r*SLC47A* R	CGGTCGGATGCCTTCTTAATCTG		
**Primers for down-regulated genes**			
r*CYP450* F	CGTCTCGTGGTGATTGTGTCGTC	*CYP450*	106
r*CYP450* R	GTGAGGTGCTTGCCAAGGAGTG		
r*LYK3* F	AGAGCAGCAGCATCATTGTCACC	*LYK3*	84
r*LYK3* R	AGAGCAGCAGCATCATTGTCACC		
r*XTH22* F	AGAGGCGGGCTGGTCAAGAC	*XTH22*	133
r*XTH22* R	AGGCGTTATTGGCGGCATTGG		
r*XTH23* F	TCCCAACGAAACAGCCAATGAGG	*XTH23*	103
r*XTH23* R	AAAGGGTGCTTGTGTCCAATCCG		
r*NAC29* F	GGAAGGCCACTGGCACAGATAAG	*NAC29*	98
r*NAC29* R	GCCCTTTGGTGGTCTACCCTTG		
r*EXO1* F	CAAGTACCATCGCCTCGCCAATC	*EXO1*	92
r*EXO1* R	GGGAGTAGGTGTCGTCGGTGAG		
r*TCP9* F	ACCACCACCAACACCAACAACAG	*TCP9*	84
r*TCP9* R	ACCACCACCAGCACCACCAG		
r*POZ4* F	ATGAGGCAATGGAGGCTCTTGTTC	*POZ 4*	137
r*POZ4* R	ATGACGAACCAGCAATTCCAGTCC		

r*UNE12* F	TACACCACCACCTCCACCATCTG	*UNE12*	143
r*UNE12* R	GATCTGTGGCTTGTCCTCGTCTTG		
r*G10H* F	AGGGAGTTCCAGGAGATGGTGTG	*G10H*	122
r*G10H* R	TACCCTGTCATCCTCTGCCTTCTG		
**Primers for house-keeping gene for normalization**			
r*Actin* F	GGCTGGATTTGCTGGAGACGATG	*Myrica rubra-Actin*	90
r*Actin* R	CCCATGCCTACCATAACACCTGTG		

Supplementary Table S2. TOP15 differentially expressed genes (DEGs）.

Gene	Function description	padj	Compared to CK(Up/Down)
At1g02270	Uncharacterized calcium-binding protein At1g02270	8.78E-250	Up
MAN 7	Mannan endo-1,4-beta-mannosidase 7	1.69E-224	Up
SCPL18	Serine carboxypeptidase-like 18	8.13E-221	Up
APG1	GDSL esterase/lipase APG	4.44E-184	Up
ICE1	Transcription factor ICE1	4.51E-134	Up
NPF2.11	Protein NRT1/ PTR FAMILY 2.11	2.10E-128	Up
GSTF11	Glutathione S-transferase F11	4.90E-124	Up
PHT1-7	Probable inorganic phosphate transporter 1-7	1.07E-123	Up
PDI1	Protein disulfide-isomerase	1.89E-116	Up
PBL16	Probable serine/threonine-protein kinase PBL16	1.21E-114	Up
ILR1	IAA-amino acid hydrolase ILR1-like 1	8.15E-111	Up
SLC47A	Protein DETOXIFICATION 24	3.74E-103	Up
KUP2	KUP system potassium uptake protein 2	7.00E-101	UP
MYB26	Transcription factor MYB26	8.35E-101	UP
CYP71A1	Cytochrome P450 71A1	1.07E-98	UP
MPK17	Mitogen-activated protein kinase kinase kinase 17	3.14E-290	Down
CYP81D1	Cytochrome P450 81D1	6.27E-264	Down
LYK3	LysM domain receptor-like kinase 3	3.59E-215	Down
XTH23	Probable xyloglucan endotransglucosylase/hydrolase protein 23	2.33E-197	Down
XTH22	Xyloglucan endotransglucosylase/hydrolase protein 22	5.25E-194	Down
NAC 29	NAC transcription factor 29	5.77E-187	Down
EXO1	Protein EXORDIUM	5.08E-181	Down
ALMT9	Aluminum-activated malate transporter 9	1.09E-180	Down
ChiC	Class V chitinase	3.80E-174	Down
TCP9	Transcription factor TCP9	3.14E-170	Down
POZ 4	BTB/POZ and TAZ domain-containing protein 4	3.66E-170	Down
ceQORH	Chloroplast envelope quinone oxidoreductase homolog	8.33E-169	Down

CLMP1	Protein CLMP1	8.69E-166	Down
UNE12	Transcription factor UNE12	1.10E-160	Down
G10H	Geraniol 8-hydroxylase	4.70E-145	Down

Supplementary Table S3. Genes related to antioxidant pathway, phenylpropanoid pathway, total phenols and flavonoids content.

Gene	Function description	padj	Compared to CK (Up/Down)
**Antioxidant pathway**			
SOD1	Superoxide dismutase [Mn], mitochondrial	0.000901611	Up
POD72	Peroxidase 72	3.40E-57	Up
POD64	Peroxidase 64	3.74E-44	Up
CAT3	Catalase isozyme 3	4.10E-75	Up
HPL1	hydroperoxide lyase	0.000612279	Up
AAO1	L-ascorbate oxidase	6.26E-103	Up
**Phenylpropionate pathway**			
EGS1	eugenol synthase	5.44E-11	Up
CSE1	caffeoyl shikimase	9.40E-29	Up
CCR1	cinnamoyl coenzyme a reductase	3.34E-48	Up
CYP84A	Ferulate-5-hydroxylase	0.034995468	Down
4CL	4-coumarate-CoA ligase	1.90E-24	Down
HCT1	O- hydroxycinnamoyltransferase	4.98E-21	Down
**Total phenols and flavonoids content**			
CYP75B1	flavonoid 3'-monooxygenase	2.57E-06	UP
VR1	vestitone reductase	4.41E-47	UP

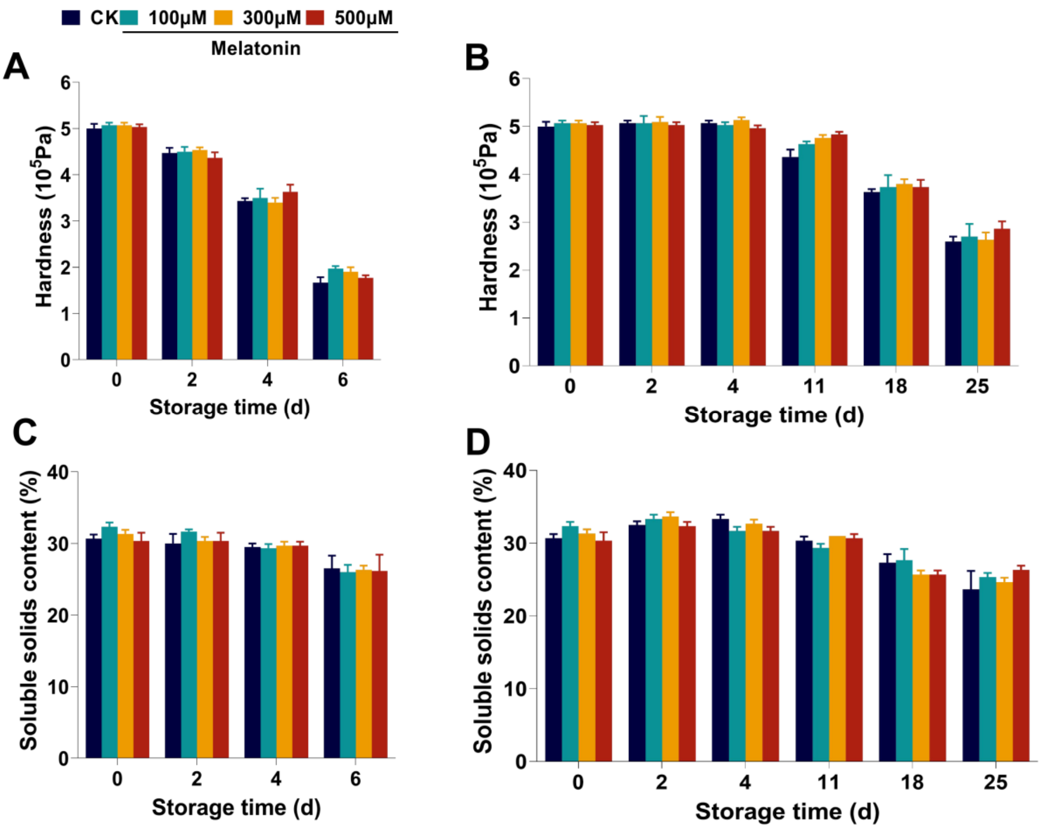

Supplementary Figure S1. Effect of pre-harvest melatonin treatment (0, 100, 300, 500 µM) on hardness and total soluble solids content (TSS) of Myrica rubra fruits during storage at room temperature and 4℃.

Pre-harvest melatonin treatment (0, 100, 300, 500µM) had no effects on the hardness **(A-B)** and TSS **(C-D)** of Myrica rubra fruits stored at room temperature for 0-6 days and 4 °C for 0-25 days (n=3 biological replicates). All results are presented as mean ± SD.

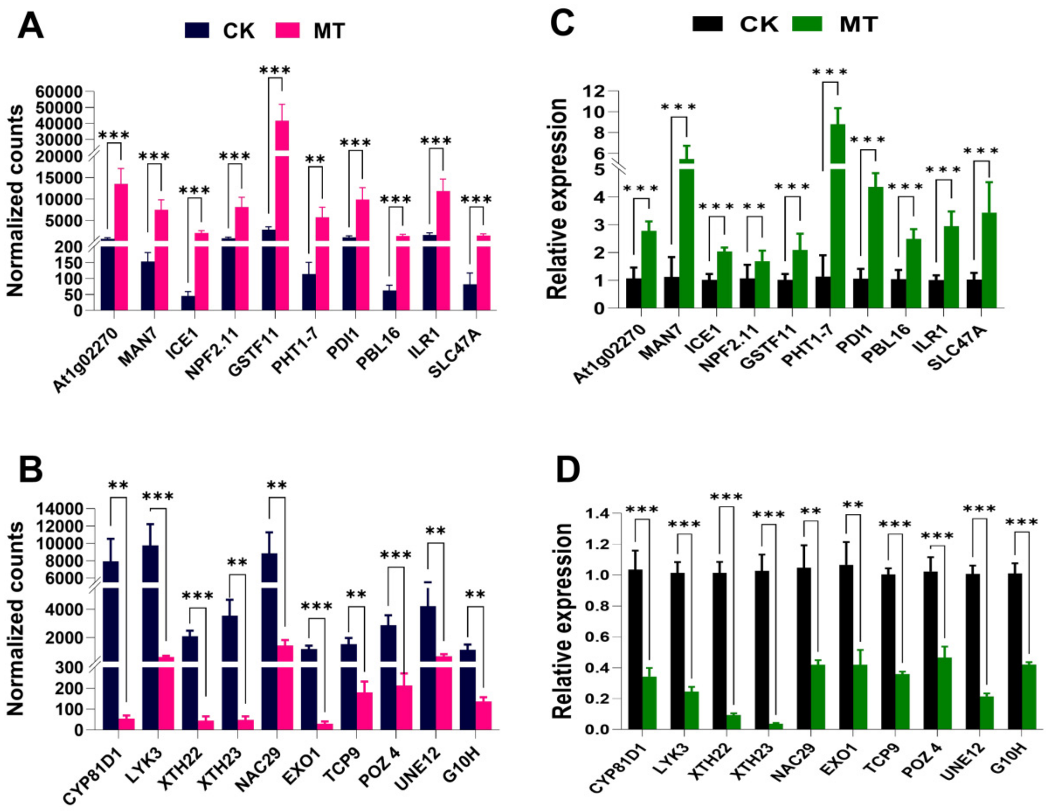

Supplementary Figure S2. Verification of the top 10 genes that showed a change in expression with pre-harvest melatonin treatments in Myrica ruba fruits. (**A-B**) Bar graphs showing the mRNA expression levels of the up-regulated (**A)** and down-regulated (**B**) top 10 genes in the Myrica ruba fruits from RNA-Seq (n=4 biological replicates). (**C-D**) Validation of the selected genes expression of up-regulated (**C**) and down-regulated (**D**) top 10 genes in the Myrica ruba fruits by using real-time quantitative PCR (n=6 biological replicates). Group differences were analyzed by two-tailed unpaired Student's t test .The data are presented as the means ± SD. **, *P*<0.01; ***, *P*<0.001.

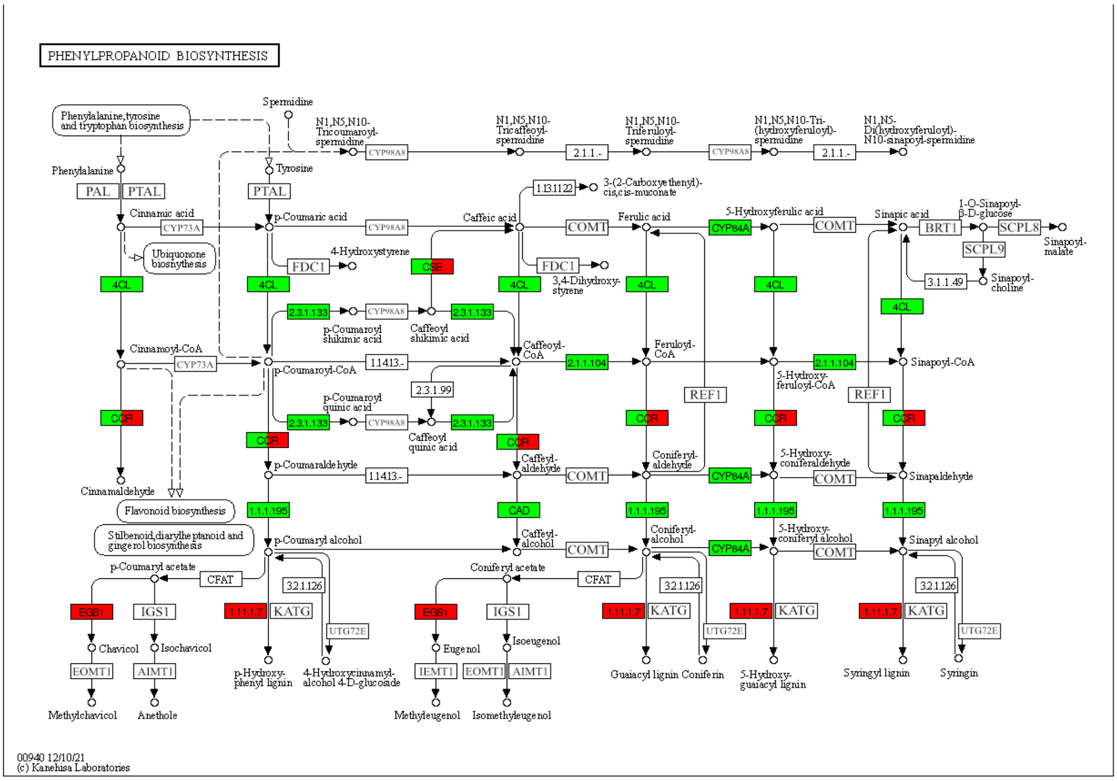

Supplementary Figure S3. The effects of pre-harvest melatonin treatment on phenylpropanoid pathway in Myrica ruba fruit.

Up-regulated genes are shown in red and down-regulated genes are shown in green.

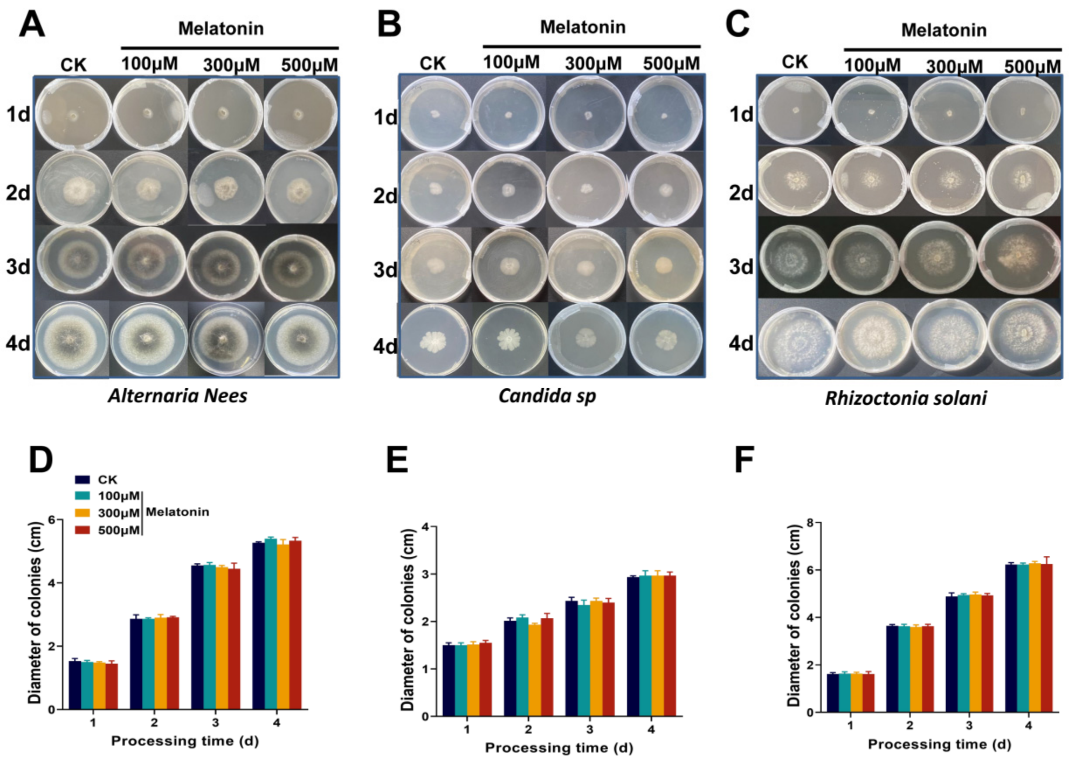

Supplementary Figure S4 Effect of the *in vitro* treatment with melatonin (0, 100, 300, 500 μM) on the morphology of fungal colonies in PDA medium and on the diameter of fungal colonies on Myrica ruba fruits.

(**A-C**) Morphology map of PDA medium fungus colonies of melatonin treatment (0, 100, 300, 500 μM) groups for *Alternaria Nees* (**A**) *Candida sp* (**B**) and *Rhizoctonia solani* (**C**) from Myrica rubra fruits stored at 1-4 days . (**D-F**) Diameter of colonies of fungus on melatonin treatment groups for *Alternaria Nees* (**D**) *Candida sp* (**E**) and *Rhizoctonia solani* (**F**) of Myrica rubra fruits stored at 1-4 days(n=3 biological replicates). All results are presented as mean ± SD.
